# Measurement of Eco-Anxiety in the Chinese Context: Development and Validation of a New Eco-Anxiety Scale Based on the Hogg Eco-Anxiety Scale

**DOI:** 10.3390/bs15070985

**Published:** 2025-07-21

**Authors:** Dawei Wang, Ziying Lu, Muze Li, Linrui Zhang, Hang Yu, Luyao Tan, Wenxu Mao, Xiuqing Qiao, Ting An, Yixin Hu

**Affiliations:** 1 Faculty of Psychology, Shandong Normal University, Jinan 250358, China; wangdw@sdnu.edu.cn (D.W.); 2023022607@stu.sdnu.edu.cn (Z.L.); 2024205046@stu.sdnu.edu.cn (M.L.); 2024205049@stu.sdnu.edu.cn (L.Z.); 2024205053@stu.sdnu.edu.cn (H.Y.); 2023010066@stu.sdnu.edu.cn (W.M.); 2023305016@stu.sdnu.edu.cn (X.Q.); 2 Shandong Provincial Key Laboratory of Brain Science and Mental Health, Jinan 250358, China; 3 Learning Department, Qingdao Malvern College, Qingdao 266106, China; 2019nicole.tan@malverncollege.cn; 4 School of Educational Science, Kashi University, Kashi 844000, China

**Keywords:** eco-anxiety, Chinese context, climate change anxiety, pro-environmental behavior

## Abstract

With the increasing complexity of ecological and environmental problems, eco-anxiety is increasingly recognized as an essential problem in China. Despite its prevalence, there is a lack of valid measurements in China. The purpose of the present study was to expand the Hogg Eco-anxiety Scale (HEAS) under the Chinese context and evaluate the psychometric attributes of the expanded scale. Specifically, a qualitative study was conducted in Study 1 (*n* = 17) to expand the HEAS in the Chinese context. Exploratory factor analysis in Study 2 (*n* = 297) and confirmatory factor analysis in Study 3 (*n* = 374) were conducted to validate the scale. The climate change anxiety scale and pro-environmental behavior scale were used to assess criterion-related validity in Study 4 (*n* = 305). Results indicated that a new eco-anxiety scale (i.e., EAS-20) including 20 items attributed to four dimension (somatic symptoms, affective symptoms, rumination, and behavioral symptoms) was developed. It showed satisfactory psychometric properties, including high internal consistency (α = 0.97) and a four-factor structure explaining 84.36% of the variance. The criterion-related validity was acceptable (0.25 ≤ *r* ≤ 0.37). The article concludes that the 20-item Eco-Anxiety Scale (EAS-20) has good psychometric properties and can be applied to measure eco-anxiety in the Chinese adult population.

## 1. Introduction

“The man of Qi was afraid that the heavens were about to fall on him” was an ancient Chinese quote in the Taoist classic *Lietzi*, which indicates the excessive anxiety and fear about losing habitable spaces due to changes in the natural environment. This quote has been used to describe the unnecessary worry of some ancestors, but today, it is no longer an unsupported phenomenon with the increased environmental change events, such as heat, hurricanes, marine pollution, and others. For instance, in a global survey, more than half of young people reported feelings of sadness, anxiety, powerlessness, or other negative emotions due to unusual environment change (Hickman et al., 2021). These negative emotions as well as distressing experiences related to the environmental change can be called eco-anxiety, which refers to a chronic fear of environmental doom (APA & ecoAmerica, 2017).

Eco-anxiety is now an emerging issue worth exploring although it was sorely neglected in the past (Pearson, 2024). Today, due to social, economic, and technological developments, people are more and more likely to directly or indirectly experience environmental change events and suffer mental distress, thereby increasing the risk of experiencing eco-anxiety. For instance, people who witnessed destroyed houses and injured people during a typhoon directly experienced concern and trauma (Pearson, 2024). More generally, experiencing extreme weather can increase people’s negative emotions and this negative effect tends to intensify over the years (Romanello et al., 2023). For people who did not personally experience environmental change events, negative environmental news reports they saw in the media also led to an increase in negative emotions and eco-anxiety (Shao & Yu, 2023). Such evidence suggests that the number of individuals experiencing eco-anxiety will continuously increase by different ways of experiencing environmental change events in the future, making eco-anxiety a pressing issue.

Regarding the consequences of eco-anxiety, it shows the characteristics of a double-edged sword. On the one hand, nearly half of the respondents reported that their worries towards ecology negatively impacted their mental health and daily functioning, such as eating, working, sleeping, and other aspects of daily life (Hickman et al., 2021), and these negative effects were magnified among individuals lacking effective coping strategies (Valero et al., 2023). On the other hand, eco-anxiety can also act as a motivator, inspiring individuals to take more environmentally friendly actions and ultimately benefiting the environment (Taylor, 2020). Overall, the double-edged sword effect of eco-anxiety indicated the importance of timely research to mitigate potential negative impacts and harness its positive benefits.

### 1.1. Review of Eco-Anxiety Measures

Designing scales to specifically measure eco-anxiety is a key step to conduct relevant research. Existing studies have used a variety of methods to assess eco-anxiety. Initially, Higginbotham et al. (2006) developed the one-factor Environmental Distress Scale (EDS), which is considered as the first scale in this field (Christodoulou et al., 2024). In addition, other researchers primarily measure eco-anxiety through assessing negative emotions about ecological issues. For instance, climate distress is measured using 12 emotional items, including concerned, tense, worried, anxious, depressed, hopeless, powerless, sad, helpless, stressed, angry, and scared (Searle & Gow, 2010). In addition, the two-dimension (personal cognitive and emotional impairment dimension, and functional impairment dimension) Climate Change Anxiety Scale (CAS) developed by Clayton and Karazsia (2020) has also been used in several studies to assess eco-anxiety though it was designed to measure climate change anxiety rather than eco-anxiety (Chung et al., 2023). What is more, the items of the CAS in the cognitive and emotional impairment dimension did not assess people’s negative emotions such as fear, anxiety, and worry. In summary, the aforementioned measurement tools just focus on some aspects of eco-anxiety (such as negative emotions and climate change anxiety) and are not designed to measure the nature of eco-anxiety, thus limiting the accuracy (validity) of measurement tools.

Different from other measurements, the Hogg Eco-anxiety Scale (HEAS) developed by Hogg et al. (2021) is a more comprehensive and valid measure of eco-anxiety, and measures individual eco-anxiety through affective symptoms, rumination, behavioral symptoms, and anxiety about personal impact dimensions. In his study, eco-anxiety is defined as experiences of anxiety related to environmental crises. Hogg et al. (2021) further stated that eco-anxiety includes not only climate change anxiety (e.g., anxious experiences related to global warming and sea level rise) but also anxiety related to multiple environmental disasters (e.g., anxious experiences related to ocean pollution and deforestation). To date, the HEAS has been successfully revised and validated for cross-cultural adaptation in several countries (e.g., Italy), and its validity and reliability have been internationally recognized (Heinzel et al., 2023; Hogg et al., 2023; Mathé et al., 2023; Rocchi et al., 2023; Sampaio et al., 2023; Türkarslan et al., 2023), but it has not yet been validated in China.

### 1.2. The Importance of Expanding the HEAS in China

The 2021 Chinese Residents’ Livelihood Opinion Survey indicated that 64.40% of respondents expressed concern about air and water pollution and other ecological problems (He, 2023; Liu, 2024), which warrants the need for its accurate assessment and early detection. However, specific and domestically applicable measures are lacking.

The HEAS provided a suitable reference for assessing public eco-anxiety in China, where eco-anxiety has been intensively experienced. However, there are still some limitations, although the HEAS has been standardized developed and widely used. First, anxiety can be shown in physical, psychological, and behavioral dimensions (Barlow, 2002), but the HEAS does not take the physiological symptoms of eco-anxiety into account. Existing literature also indicates that people with eco-anxiety may experience panic attacks, insomnia, repeated negative thinking, a loss of appetite, or shaking, suggesting that individuals with eco-anxiety may also exhibit somatic symptoms (Dockett, 2019; Hickman, 2020; Mulligan et al., 2023). Therefore, adding a new dimension, somatic symptoms, to capture more manifestations of eco-anxiety is necessary.

Moreover, considering that the HEAS has been tested mostly in samples in an individualistic culture, while China is a country with a collectivist culture, it seems necessary to supplement the specific manifestations in the original dimensions. According to the social information processing (SIP) model and the integrated model of emotional processes and cognition, when individuals think about the global environmental crisis, they make cognitive interpretations (e.g., rumination), have emotional responses (e.g., emotional symptoms), and then act (Crick & Dodge, 1994; Lemerise & Arsenio, 2000). Relevant literature suggests that individuals experiencing haze anxiety exhibit cognitive processes (e.g., fearing that the haze poses a potential threat to their health), emotional symptoms (e.g., feeling irritable and depressed), and avoidance behaviors (e.g., unwillingness to go to work or school, or a desire to leave the city immediately) (Li & Tao, 2021). These characteristics are present in Chinese people but are not included in the Hogg Eco-anxiety Scale. Therefore, this study also intended to expand the content of the rumination, emotional symptoms, and behavioral symptoms dimensions through semi-structured interviews to capture the various manifestations of eco-anxiety more comprehensively.

### 1.3. The Relationship of Eco-Anxiety with Climate Change Anxiety and Pro-Environmental Behavior

In order to further verify the criterion-related validity of the expanded scale, this study adopted climate change anxiety and pro-environmental behavior as the criteria. Climate change anxiety reflects the concern and fear about climate change and its potential impact, and constitutes a significant aspect of eco-anxiety (Clayton & Karazsia, 2020; Hogg et al., 2021; Parmentier et al., 2024), so we speculate that eco-anxiety is positively correlated with climate change anxiety.

Secondly, implementing pro-environmental behavior is one of the means for individuals to cope with eco-anxiety (Pihkala, 2020). Pro-environmental behavior refers to the commission of acts that benefit the natural environment and the omission of acts that harm it (Lange & Dewitte, 2019). There is evidence that eco-anxiety is significantly and positively correlated with individuals’ environmentally friendly behaviors, and it can consistently predict such behaviors (Hogg et al., 2024; Pavani et al., 2023). Specifically, individuals experiencing eco-anxiety tend to adopt positive coping strategies by engaging in environmental protection activities and taking practical actions (e.g., reusing goods, recycling goods, etc.) to alleviate their negative psychological responses to environmental changes (Chung et al., 2023; Kabasakal-Cetin, 2023; Parreira & Mouro, 2023; Valero et al., 2023). In light of the aforementioned evidence, we also speculate that there is a positive correlation between eco-anxiety and pro-environmental behavior.

### 1.4. The Present Study

Overall, as more and more people are experiencing and deeply affected by eco-anxiety, there is an urgent need for a validated questionnaire. Based on possible physical manifestations of anxiety, the SIP model and the HEAS, the main purpose of this study is to assess the psychometric properties of the expanded scale. Specifically, this study expanded the HEAS through semi-structured interviews in Study 1, and then examined the structural validity, reliability, and criterion-related validity of this scale in Studies 2, 3, and 4, respectively.

## 2. Study 1: Qualitative Research on the Concept of Eco-Anxiety

### 2.1. Research Objective

To analyze the characteristics and dimensions of eco-anxiety through theoretical analysis and semi-structured interviews and form an eco-anxiety questionnaire suitable for Chinese adults.

### 2.2. Participants

A total of 17 adults from universities, enterprises, hospitals, were interviewed (females = 10; age range = 22–55 years; *M*_age_ = 34.47 years) to capture their perspectives on eco-anxiety.

### 2.3. Interview Syllabus

Referring to the somatic symptoms related to anxiety disorder and also included in the SCL-90 (APA, 2013; Derogatis et al., 1973), and based on the original dimensions of the HEAS (emotional symptoms, rumination, behavioral symptoms, and anxiety about personal impact) and relevant literature, a semi-structured interview protocol was developed and evaluated by experts. The content of the protocol included (1) the connotation of eco-anxiety; (2) the impacts of ecological deterioration on individuals; (3) the experiences of eco-anxiety; (4) the manifestations of individuals experiencing eco-anxiety; (5) the influencing factors of eco-anxiety; and (6) strategies for mitigating individuals’ eco-anxiety.

### 2.4. Procedure and Data Analysis

The interviews were conducted in a private setting, and participants signed informed consent forms before the interviews, which provided information on the purpose, content, recording, and confidentiality of the study. Each interview was conducted according to the established protocol and lasted about 30 min with continuous audio recording throughout. These recorded interviews were transcribed into Chinese text and analyzed by four psychology professionals using Nvivo 11. Specifically, textual information relevant to each dimension was identified from the interview transcripts to generate open codes. Subsequently, the textual information was synthesized to form axial codes. Finally, core codes were developed based on the open and axial codes, culminating in the construction of an item bank.

### 2.5. Results

A total of 655 open codes, 26 axial codes, and 5 core codes were ultimately formulated. The axial codes “tinnitus” (2 instances) and “dizziness” (2 instances) were categorized under “somatic symptoms”. The axial codes “feeling nervous, anxious or on edge” (69 instances), “not being able to stop or control worrying” (23 instances), “worrying too much” (21 instances), “feeling afraid” (22 instances), “feeling angry” (65 instances), and “others” (96 instances) were categorized under “emotional symptoms”. The axial codes “unable to stop thinking about future climate change and other global environmental problems” (18 instances), “unable to stop thinking about past events related to climate change” (32 instances), “unable to stop thinking about losses to the environment” (2 instances), “unable to stop thinking about the impact on current life” (32 instances), “unable to stop thinking about the impact on future generations” (36 instances), “unable to stop thinking about the causes of the environmental crisis” (12 instances), “unable to stop thinking about measures to change the environmental situation” (19 instances), and “unable to stop thinking about the relationship between humans and nature” (17 instances) were categorized under “rumination”. The axial codes “difficulty sleeping” (11 instances), “difficulty working and/or studying” (12 instances), “difficulty controlling repetitive behaviors” (12 instances), “avoidance behaviors” (31 instances), “maladaptation” (5 instances), “eating quantity” (7 instances), and “eating choices” (26 instances) were categorized under “behavioral symptoms”. The axial codes “feeling anxious about the impact of your personal behaviors on the earth” (10 instances), “feeling anxious about personal responsibility to help address environmental problems” (19 instances), and “feeling anxious about one’s inability to help solve environmental problems” (54 instances) were categorized under “anxiety about personal impact”.

After completing the coding, the inter-coder reliability assessment was conducted. The Kappa coefficients for each node were calculated by comparing the codes assigned by two coders. The final results indicated that all Kappa coefficients were greater than 0.4 (see Table 1), demonstrating good coding consistency.

### 2.6. Summary

After the relevant items were formed, four professors specializing in environmental psychology and clinical and counseling psychology were invited to discuss and conduct expert appraisals. During the discussion, items with unclear meaning, similar content, and grammatical errors were deleted or modified to form the 25-item version of the Hogg Eco-Anxiety Scale (HEAS-25). Specifically, HEAS-25 involved the somatic symptoms dimension (four items), the emotional symptoms dimension (five items), the rumination dimension (eight items), the behavioral symptoms dimension (five items), and the anxiety about personal impact dimension (three items). This scale used a four-point scoring system, where zero represents “not at all,” one represents “several of the days”, two represents “over half the days” and three represents “nearly every day”.

## 3. Study 2: Exploratory Factor Analysis of the HEAS-25

### 3.1. Research Objective

To remove items that do not meet the requirements and derive the structure of the eco-anxiety scale according to item analysis and exploratory factor analysis, respectively, and ultimately form the final version of the scale.

### 3.2. Participants

Participants in this study were a total of 297 adults (52.2% females, *n* = 155), aged 18–66 years (*M* = 21.00 years, *SD* = 5.14), who were recruited from the eastern region (*n* = 170, 57.2%), central region (*n* = 28, 9.4%), and western region (*n* = 99, 33.3%) of China. This study collected informed consent from all participants and complied with the ethical standards of the 1964 Helsinki Declaration and the academic committee of Shandong Normal University.

### 3.3. Measures

The 25-item version of the Hogg Eco-Anxiety Scale (HEAS-25). The HEAS-25 consists of 25 items. All items were rated on a 4-point Likert scale (0 = not at all, 3 = nearly every day). A sample item was “Experiencing tinnitus.” The higher the score, the greater the severity of eco-anxiety. The Cronbach’s α for this scale was 0.98.

### 3.4. Procedure and Data Analysis

After obtaining informed consent from the participants, the electronic questionnaire was distributed and collected by a professional platform. Data were analyzed using SPSS 22.0.

### 3.5. Results

#### 3.5.1. Item Analysis

Item–total correlation analysis and item discrimination analysis were conducted. Results indicated that all items met the required standard, with item-total correlations exceeding 0.4 (see Table 2). Referring to Kelley’s (1939) suggestion on the item discrimination analysis, an independent samples *t*-test between the high eco-anxiety group (top 27%) and low eco-anxiety group (bottom 27%) was conducted and revealed a significant difference (*t* = 20.104, *p* < 0.001, Cohen’s *d* = 3.189), indicating that the HEAS-25 demonstrated good discrimination ability.

#### 3.5.2. Exploratory Factor Analysis and Reliability Analysis

Principal component analysis (PCA) was used as the extractor method to extract five factors. As for the method of factor rotation, aligned with the suggestions of previous studies and the goals of scale development, Varimax (one of the orthogonal rotation methods) was chosen as the rotation method, which focuses more on the independence of each factor rather than cross-loadings, and can ensure the clarity, interpretability, and invariance of the factors (Alordiah & Chenube, 2023; Castro et al., 2015; Ricolfi & Testa, 2021). The results of Bartlett’s test showed that χ^2^ was 9808.713 (*df* = 300, *p* < 0.001), and the Kaiser–Meyer–Olkin (KMO) value was 0.962, indicating that the data were suitable for subsequent analysis. Factor loadings were presented in Table 3 and the exclusion criteria were as follows: (1) items with communalities less than 0.3; (2) factor loading below 0.45 (Comrey & Lee, 1992); and (3) factor loadings attributed to more than one factor. Based on these criteria, items T21, T22, T23, T24, and T25 were removed, leaving 20 items attributed to 4 factors.

Principal component analysis was conducted again on the remaining 20 items to extract 4 factors and Varimax was used to rotate factors (see Table 4). The results of Bartlett’s test showed that χ^2^ was 7355.271 (*df* = 190, *p* < 0.001), and the KMO value was 0.955, reaching statistical significance and explaining 84.36% of the variance. Factor 1 was named ‘rumination’ and consisted of 8 items (T10–T17) with factor loadings ranging from 0.76 to 0.85, which explained 67.27% of the variance. Factor 2 was named ‘emotional symptoms’ and consisted of 5 items (T5–T9) with factor loadings ranging from 0.70 to 0.78, which explained 8.23% of the variance. Factor 3 was named ‘somatic symptoms’ and consisted of 4 items (T1–T4) with factor loadings ranging from 0.76 to 0.83, which explained 5.35% of the variance. Factor 4 was named ‘behavioral symptoms’ and consisted of 3 items (T18–T20) with factor loadings ranging from 0.66 to 0.72, which explained 3.51% of the variance. In the 20-item eco-anxiety scale, the Cronbach’s α for the whole scale, somatic symptoms dimension, emotional symptoms dimension, rumination dimension, and behavioral symptoms dimension was 0.97, 0.94, 0.93, 0.98, and 0.92, respectively.

### 3.6. Summary

A new eco-anxiety scale (i.e., The 20-item Eco-Anxiety Scale, EAS-20) was developed based on the Hogg Eco-Anxiety Scale. It includes 20 items (10 original items from HEAS and 10 newly added items) attributed to four factors, which were named as follows based on the content covered by the items: rumination (Factor 1 with eight items), emotional symptoms (Factor 2 with five items), somatic symptoms (Factor 3 with four items), and behavioral symptoms (Factor 4 with three items). The EAS-20 demonstrated high internal consistency with a Cronbach’s α of 0.97.

## 4. Study 3: Confirmatory Factor Analysis of the EAS-20

### 4.1. Participants

Participants in this study were a total of 374 adults (47.6% females, *n* = 178), aged 18 to 53 years (*M* = 21.23 years, *SD* = 5.95), who were recruited from the eastern region (*n* = 228, 61.0%), central region (*n* = 51, 13.6%), and western region (*n* = 95, 25.4%) of China. This study collected the informed consent of all participants and complied with the ethical standards of the 1964 Helsinki Declaration and the academic committee of Shandong Normal University.

### 4.2. Measures

The 20-item Eco-Anxiety Scale (EAS-20). The EAS-20 covers four dimensions: somatic symptoms (4 items), affective symptoms (5 items), rumination (8 items), and behavioral symptoms (3 items). A sample item was “Experiencing tinnitus.” All items were rated on a 4-point Likert scale (0 = not at all, 3 = nearly every day). The higher the score, the greater the severity of eco-anxiety. In this study, the Cronbach’s α for this scale was 0.97.

### 4.3. Procedure and Data Analysis

After obtaining informed consent from the participants, trained professionals distributed and collected the electronic questionnaires. All analyses were conducted using SPSS 22.0 and Mplus 8.0.

### 4.4. Results

Based on the results of the EFA, the EAS-20 was set to a four-dimensional structure, a confirmatory factor analysis (CFA) was performed using structural equation modelling. The χ^2^/*df* value, Tucker–Lewis Index (TLI), comparative fit index (CFI), standardized root mean square residual (SRMR), and root mean square error of approximation (RMSEA) were selected as indices to assess the model fit. The following criteria were recognized as the threshold of an acceptable model fit: χ^2^/*df* < 3 (Hu & Bentler, 1999), RMSEA < 0.08 (Byrne et al., 1989), CFI ≥ 0.95 (Hu & Bentler, 1999), TLI > 0.90 (Kline, 2011), and SRMR < 0.08 (Hu & Bentler, 1999). The results revealed that all fit indices satisfied the criteria (χ^2^/*df* = 2.20, RMSEA = 0.06, CFI = 0.95, TLI = 0.94, and SRMR = 0.04) and the fit was significantly superior to alternative models (see Table 5). These results provided further support for the four-dimensional structure of the EAS-20 and confirmed its structural validity.

### 4.5. Summary

The results of the CFA were consistent with the original hypothesis, indicating that the 20-item Eco-Anxiety Scale (EAS-20) included four dimensions: somatic symptoms, emotional symptoms, rumination, and behavioral symptoms, with a Cronbach’s α of 0.97. Therefore, the EAS-20 has good structural validity and reliability, making it suitable for use in future research.

## 5. Study 4: Criterion-Related Validity Testing of the EAS-20

### 5.1. Participants

Participants in this study were a total of 305 adults (48.9% females, *n* = 149), aged 18 to 69 years (*M* = 21.16 years, *SD* = 6.59), who were recruited from the eastern region (*n* = 197, 64.6%), central region (*n* = 42, 13.8%), and western region (*n* = 66, 21.6%) of China. This study collected informed consent from all participants and complied with the ethical standards of the 1964 Helsinki Declaration and the academic committee of Shandong Normal University.

### 5.2. Measures

The 20-item Eco-Anxiety Scale (EAS-20). The scale was the same as in Study 3. In this study, the Cronbach’s α for this scale was 0.97.

The Climate Change Anxiety Scale (CCAS). The 9-item scale developed by Clayton and Karazsia (2020) and revised by Guo and Lv (2023) was used to measure climate change anxiety, including 2 dimensions: personal cognitive and emotional impairment and functional impairment. A sample item is, “Thinking about climate change makes it difficult for me to concentrate.” All items were scored on a 5-point Likert scale (1 = never, 5 = always) with higher scores indicating higher levels of climate anxiety. In this study, the Cronbach’s α of this scale was 0.98.

Pro-environmental Behavior Scale (PEBS). Nine items appropriate for the Chinese context were selected from the scale developed by Schultz (2000), which includes 12 past green behaviors to measure individuals’ pro-environmental behavior. Participants were asked to indicate the frequency with which they did the following activities in the past year. A sample item was “Conserved gasoline by walking or bicycling.” A 5-point Likert scale (1 = never, 5 = always) was used, with higher scores indicating a higher frequency of engagement in pro-environmental behavior. In this study, the Cronbach’s α of the scale was 0.94.

### 5.3. Procedure and Data Analysis

After obtaining informed consent from the participants, the electronic questionnaires were distributed and collected by professional staff. Data were analyzed using SPSS 22.0.

### 5.4. Results

The correlations between EAS-20, CCAS, and PEBS are shown in Table 6. The results indicated that EAS-20 was significantly positively correlated with CCAS (*r* = 0.37, *p* < 0.001) and PEBS (*r* = 0.25, *p* < 0.001), which suggests that the EAS-20 has good criterion-related validity.

### 5.5. Summary

Consistent with the original hypothesis, a positive link between climate change anxiety and eco-anxiety was found, indicating that individuals experiencing higher levels of climate change anxiety also tended to have heightened concerns about the whole ecosystem. The positive relationship between eco-anxiety and pro-environmental behavior meant individuals who were more anxious about ecological issues were more likely to engage in behaviors that are beneficial to the environment. These findings suggest that the EAS-20 has good criterion-related validity.

## 6. Discussion

The present study expanded the HEAS, and formed and validated a new eco-anxiety scale (i.e., EAS-20) containing ten original items from the HEAS and ten newly added items. The results of EFA and CFA found evidence that the EAS-20 has a stable internal structure, consisting of four subscales assessing somatic symptoms, affective symptoms, rumination, and behavioral symptoms, and showed good internal consistency.

Different from the HEAS (Hogg et al., 2021), the EAS-20 deleted the anxiety about personal impact dimension and added a somatic symptoms dimension. Specifically, the anxiety about personal impact dimension was deleted due to cultural differences. Compared to individuals in individualistic cultural backgrounds (e.g., New Zealand and Australia; Hogg et al., 2021, 2023), who exhibit higher personal responsibility for environmental action and impact, individuals in collectivist cultures (e.g., China) emphasize collective responsibility. More specifically, they tend to adopt a collective perspective, believing that their individual impact is insufficient to change the environment and that collective impact plays a more important role in causing adverse environmental consequences or achieving environmental protection goals. Hence, the anxiety about personal impact dimension was not an appropriate and valid aspect to differentiate individuals with eco-anxiety in the Chinese context. In addition, consistent with research on (sub) clinical forms of anxiety (APA, 2013), the somatic symptoms dimension was incorporated into the EAS-20, confirming that symptoms like tinnitus and headache are significant indicators of eco-anxiety. Furthermore, biological commonalities (e.g., similar brain region activity) could account for the similar somatic reactions across various anxiety types (Zhang et al., 2011).

More manifestations related to eco-anxiety were also added to emotional symptoms and rumination dimensions. First, the item “Becoming easily annoyed or irritable” was excluded in the HEAS (Hogg et al., 2021), but our findings suggest that anger (T9) is a hallmark of eco-anxiety among Chinese individuals in the EAS-20. Relevant research pointed out that negative information about the global environmental crisis triggered not only personal concern and fear (Gómez et al., 2022; Leger-Goodes et al., 2022; Pihkala, 2022), but also anger (Leger-Goodes et al., 2022). This may be because environmental uncertainty can lead to a lack of control (Mittal & Griskevicius, 2014), which makes people experience fear and subsequently triggers anger as a mechanism to bolster personal control and achieve certainty (Lerner & Keltner, 2000). Second, more items about rumination were supplemented. Negative information, such as environmental crises and their adverse impacts, would captivate the attention of individuals with eco-anxiety due to negative attention bias (Gregory et al., 2019; Hou et al., 2021), leading to repeated thinking about attribution, adverse consequences, and coping methods related to the environmental crisis. Meanwhile, considering the long-term orientation within collectivism (Hofstede, 1980), people always adopt a sustainable attitude towards development, so items focusing on the well-being of the next generation (T14) and the human–nature relationship (T17) were reasonable.

With respect to the criterion-related validity, EAS-20 showed significant and positive correlations with CCAS and PEBS. Climate change anxiety is encompassed within the experience of eco-anxiety (Hogg et al., 2021; Rocchi et al., 2023), and the positive correlation between the EAS-20 and CCAS indicated good criterion-related validity, which was consistent with previous research (Hogg et al., 2021; Rocchi et al., 2023). Investigating the behavioral consequences of eco-anxiety, such as pro-environmental behavior, is another important line of research. Existing studies suggests that eco-anxiety is a potential driver of pro-environmental behavior (Rocchi et al., 2023; Türkarslan et al., 2023). The positive correlation between the EAS-20 and PEBS in our study further supported this view, aligning with the main findings of Hogg et al. (2024) and confirming the criterion-related validity of the EAS-20. However, it is worth noticing that whether an individual’s eco-anxiety can be transferred into actual pro-environmental behavior may be influenced by supporting context (such as organizations’ undertaking of environmental responsibility; Wang et al., 2023) and individual’s self-efficacy in performing green actions (Wang et al., 2025).

Overall, the present study has several advantages in terms of applicability, explanatory power, and practical use. In terms of applicability, the EAS-20, by excluding the anxiety about personal impact dimension, aligns better with Chinese cultural contexts compared to the HEAS. In terms of explanatory power, the factor loadings of the EAS-20 ranged from 0.66 to 0.85 and explained more variance (84.36%) than the HEAS (82.15%) by adding the somatic symptoms dimension and more manifestations in other existing dimensions. Additionally, in terms of practical use, the EAS-20, with its good psychometric characteristics, is the first eco-anxiety scale in China, which provides a reliable measurement tool for future research and interventions on eco-anxiety. Specifically, it can be used to study the mechanisms of antecedents and outcomes of eco-anxiety, as well as to identify individuals who are experiencing eco-anxiety.

However, this study still has some limitations. First, our study validated the EAS-20 in a general population. However, future studies should consider clinical groups with potentially different manifestations. In addition, the results indicate that the anxiety about personal impact dimension was not suitable for China’s collectivist context. Hence, further exploration is needed into how the anxiety about societal impact plays a role different from anxiety of personal impact, as also highlighted by Heinzel et al. (2023). Finally, the data in the present study were self-reported and some objective indicators (e.g., cortisol level) need to be included in future research for in-depth study.

## 7. Conclusions

This study formed and validated a new eco-anxiety scale (i.e., EAS-20) based on the Hogg Eco-Anxiety Scale in the Chinese context. A four-factor structure—somatic symptoms, emotional symptoms, rumination, and behavioral symptoms—was identified, which was distinct from the HEAS. The EAS-20 showed high internal consistency, sufficient structural validity, and good criterion-related validity, suggesting that the EAS-20 can be used in future studies focusing on the assessment, detection, and intervention of eco-anxiety in China.

## Figures and Tables

**Table 1:** Kappa coefficients for each node.

	Kappa (κ)
Somatic symptoms	0.43
Tinnitus	1.00
Dizziness	1.00
Affective symptoms	0.69
Feeling nervous, anxious or on edge	0.47
Not being able to stop or control worrying	0.52
Worrying too much	0.60
Feeling afraid	0.41
Feeling angry	0.75
Others	0.80
Rumination	0.92
Unable to stop thinking about future climate change and other global environmental problems	0.98
Unable to stop thinking about past events related to climate change	0.96
Unable to stop thinking about losses to the environment	0.90
Unable to stop thinking about the impact on current life	0.88
Unable to stop thinking about the impact on future generations	0.94
Unable to stop thinking about the causes of the environmental crisis	0.97
Unable to stop thinking about measures to change the environmental situation	0.92
Unable to stop thinking about the relationship between humans and nature	0.79
Behavioral symptoms	0.84
Difficulty sleeping	0.48
Difficulty working and/or studying	0.98
Difficulty controlling repetitive behaviors	0.80
Avoidance behaviors	0.81
Maladaptation	0.78
Eating quantity	0.49
Eating choices	0.80
Anxiety about personal impact	0.85
Feeling anxious about the impact of your personal behaviors on the earth	0.88
Feeling anxious about your personal responsibility to help address environmental problems	0.82
Feeling anxious that your personal behaviors will do little to help fix the problem	0.86

**Table 2:** Item–total correlations of the HEAS-25 (*r_it_*).

Item	*r_it_*
T1 Experiencing tinnitus.	0.73 ***
T2 Experiencing headache.	0.74 ***
T3 Experiencing dizziness.	0.77 ***
T4 Experiencing nausea or stomach discomfort.	0.75 ***
T5 Feeling nervous, anxious or on edge.	0.79 ***
T6 Not being able to stop or control worrying.	0.82 ***
T7 Worrying too much.	0.77 ***
T8 Feeling afraid.	0.79 ***
T9 Feeling angry.	0.75 ***
T10 Unable to stop thinking about future climate change and other global environmental problems.	0.89 ***
T11 Unable to stop thinking about past events related to climate change	0.86 ***
T12 Unable to stop thinking about losses to the environment.	0.88 ***
T13 Unable to stop thinking about the impact on current life.	0.87 ***
T14 Unable to stop thinking about the impact on future generations.	0.85 ***
T15 Unable to stop thinking about the causes of the environmental crisis.	0.85 ***
T16 Unable to stop thinking about measures to change the environmental situation.	0.86 ***
T17 Unable to stop thinking about the relationship between humans and nature.	0.86 ***
T18 Difficulty sleeping.	0.83 ***
T19 Difficulty enjoying social situations with family and friends.	0.82 ***
T20 Difficulty working and/or studying.	0.81 ***
T21 Difficulty controlling repetitive behaviors, including but not limited to hand washing and checking rituals.	0.88 ***
T22 Difficulty controlling the desire to escape from a damaged environment.	0.89 ***
T23 Feeling anxious about the impact of your personal behaviors on the earth.	0.87 ***
T24 Feeling anxious about your personal responsibility to help address environmental problems.	0.88 ***
T25 Feeling anxious that your personal behaviors will do little to help fix the problem.	0.86 ***

Note: *** *p* < 0.001.

**Table 3:** Principal component analysis (25 items).

Item	1	2	3	4	5	Communalities
T10 Unable to stop thinking about future climate change and other global environmental problems.	0.78					0.88
T11 Unable to stop thinking about past events related to climate change.	0.78					0.85
T12 Unable to stop thinking about losses to the environment.	0.76					0.86
T13 Unable to stop thinking about the impact on current life.	0.80					0.88
T14 Unable to stop thinking about the impact on future generations.	0.75					0.83
T15 Unable to stop thinking about the causes of the environmental crisis.	0.82					0.87
T16 Unable to stop thinking about measures to change the environmental situation.	0.83					0.89
T17 Unable to stop thinking about the relationship between humans and nature.	0.80					0.86
T21 Difficulty controlling repetitive behaviors, including but not limited to hand washing and checking rituals.	0.47			0.62		0.85
T22 Difficulty controlling the desire to escape from a damaged environment.	0.52			0.50		0.83
T23 Feeling anxious about the impact of your personal behaviors on the earth.	0.51				0.51	0.87
T24 Feeling anxious about your personal responsibility to help address environmental problems.	0.57				0.54	0.93
T25 Feeling anxious that your personal behaviors will do little to help fix the problem.	0.55				0.56	0.89
T5 Feeling nervous, anxious, or on edge.		0.70				0.79
T6 Not being able to stop or control worrying.		0.73				0.85
T7 Worrying too much.		0.76				0.83
T8 Feeling afraid.		0.69				0.80
T9 Feeling angry.		0.76				0.79
T1 Experiencing tinnitus.			0.80			0.83
T2 Experiencing headache.			0.76			0.81
T3 Experiencing dizziness.			0.82			0.89
T4 Experiencing nausea or stomach discomfort.			0.83			0.88
T18 Difficulty sleeping.				0.64		0.80
T19 Difficulty enjoying social situations with family and friends.				0.74		0.87
T20 Difficulty working and/or studying.				0.71		0.83

Note: Factor loadings below 0.45 were omitted.

**Table 4:** Principal component analysis (20 items).

Item	1	2	3	4	Communalities
T10 Unable to stop thinking about future climate change and other global environmental problems.	0.80				0.88
T11 Unable to stop thinking about past events related to climate change.	0.79				0.84
T12 Unable to stop thinking about losses to the environment.	0.78				0.86
T13 Unable to stop thinking about the impact on current life.	0.82				0.88
T14 Unable to stop thinking about the impact on future generations.	0.76				0.81
T15 Unable to stop thinking about the causes of the environmental crisis.	0.84				0.87
T16 Unable to stop thinking about measures to change the environmental situation.	0.85				0.89
T17 Unable to stop thinking about the relationship between humans and nature.	0.82				0.86
T5 Feeling nervous, anxious or on edge.		0.71			0.79
T6 Not being able to stop or control worrying.		0.75			0.84
T7 Worrying too much.		0.78			0.82
T8 Feeling afraid.		0.70			0.79
T9 Feeling angry.		0.77			0.79
T1 Experiencing tinnitus.			0.81		0.83
T2 Experiencing headache.			0.76		0.80
T3 Experiencing dizziness.			0.83		0.89
T4 Experiencing nausea or stomach discomfort.			0.83		0.88
T18 Difficulty sleeping.				0.66	0.83
T19 Difficulty enjoying social situations with family and friends.				0.71	0.86
T20 Difficulty working and/or studying.				0.72	0.86

Note: Factor loadings below 0.45 were omitted.

**Table 5:** Results of CFA fit indices.

Model	χ^2^/*df*	RMSEA	CFI	TLI	SRMR
Four-factor Model (Predictive Model)	2.20 ***	0.06	0.95	0.94	0.04
Three-factor Model	3.25 ***	0.08	0.90	0.89	0.06
Two-factor Model	4.74 ***	0.10	0.83	0.81	0.07
One-factor Model	6.13 ***	0.12	0.77	0.74	0.08

Note: *** *p* < 0.001; four-factor model: A, B, C, and D; three-factor model: A, B, and C+D; two-factor model: A and B+C+D; and one-factor model: A+B+C+D.

**Table 6:** Correlations between EAS-20, CCAS, and PEBS.

	*M*	*SD*	1	2	3
1 EAS-20	0.41	0.54	-		
2 CCAS	2.13	0.99	0.37 ***	-	
3 PEBS	2.66	0.93	0.25 ***	0.59 ***	-

Note: N = 305. *** *p* < 0.001.

## Data Availability

The data that support the findings of this study are available from the corresponding author upon reasonable request.
